# Drunkorexia: is it really “just” a university lifestyle choice?

**DOI:** 10.1007/s40519-020-01051-x

**Published:** 2020-10-30

**Authors:** Bethany Leigh Griffin, Katharina Sophie Vogt

**Affiliations:** Department of Psychology, The University of Huddersfield, Queensgate, Huddersfield, HD1 3DH UK

**Keywords:** Drunkorexia, Alcohol use, Substance use, Compensatory behaviours, Disordered eating

## Abstract

**Purpose:**

The current study investigated the prevalence of compensatory behaviours (caloric restriction, increased exercise and bulimic tendencies) in response to alcohol consumption (also known as Drunkorexia) in students, non-students and previous students, as well as beginning to understand the presence of possible predictors of these behaviours (body esteem, sensation seeking).

**Methods:**

A volunteer sample of students, non-students and previous students (*n* = 95) completed the Compensatory Eating and Behaviours in Response to Alcohol Consumption Scale, a questionnaire which measures overall Drunkorexia engagement. The participants also completed the Body Esteem Scale for Adolescents and Adults Scale (BESAA) and the Brief Sensation Seeking Scale (BSSS) to investigate predictors of Drunkorexia.

**Results:**

The results indicated that there was no significant difference in Drunkorexia engagement and behaviours between students, non-students and previous students. It was also found that both low body esteem and high sensation seeking tendencies were significant predictors of Drunkorexia; specifically, the appearance esteem factor of the BESAA and the disinhibition factor of the BSSS.

**Conclusions:**

Findings suggest that Drunkorexia is also present outside of student populations, and therefore, future interventions and research should include non-students in samples. In addition, findings support the idea that Drunkorexia cannot be classified solely as an eating disorder or a substance abuse disorder. As a result of this, further research should be conducted to fully understand why this complex behaviour exists.

**Evidence-based medicine level:**

III (Evidence obtained from case-control analytic study)

**Electronic supplementary material:**

The online version of this article (10.1007/s40519-020-01051-x) contains supplementary material, which is available to authorized users.

## Introduction

Alcohol is a widely used, socially acceptable ‘drug’ that alters a person’s perception when consumed [1]. Its potential detrimental effects to the health of the individual, society and health care systems are widely described. On an individual level, heavy alcohol consumption is associated with effects on the brain, leading to reduced abilities of, for example, memory and executive function [2]. On a societal and health care system level, the cost of excessive alcohol consumption is extensively documented: for example, in 2017/2018, there were 1.2 million hospital admissions as a result of excess alcohol consumption in the UK leading to an estimated yearly cost of 3.5 billion pounds a year [3]. A population that is especially associated with excessive alcohol consumption are university students; with excessive alcohol consumption being viewed as key to the university student lifestyle [4–6].

The effects of alcohol on the brain are largely dependent on blood alcohol concentration; with higher levels creating greater impairments in attention, vigilance, problem solving and reaction time [7–9]. A moderating factor for blood alcohol concentration is food intake. Alcohol is absorbed into the blood stream through the stomach, meaning when alcohol is consumed with no prior food intake, blood alcohol concentration is elevated due to much quicker absorption [10]. This can thus exacerbate the effects of alcohol significantly.

The relationship between alcohol and food intake has long been investigated. Disordered eating combined with alcohol abuse has been highlighted for both men and women [11, 12]. This link is thought to be bi-directional, meaning that as alcohol consumption increases, food intake decreases; and vice versa [13]. Hypothesised, potential motivating factors for these behaviours are feeling the effects of alcohol more quickly or limiting calories intake via food to balance the caloric value in alcohol [14].

This use of restricted eating in response to alcohol consumption has been labelled Drunkorexia [15]. In this context, reduced eating is seen as both an inappropriate compensatory behaviour to avoid weight gain [16], and a food and alcohol disturbance [11]. While some limited research has been conducted investigating the prevalence of these behaviours, as well as underlying motivations and reasonings; many questions are left unanswered [17].

In recent years, there has been an increase in research interest and attention to Drunkorexia. Regarding prevalence, Roosen and Mills [18], for example, showed that 42% of their Canadian undergraduate sample were concerned with the caloric content of the alcohol they are consuming, and 37% did restrict their food before alcohol consumption. Furthermore, Knight et al. [19] showed that of their Australian all-female sample, 64% of participants used compensatory behaviours in response to alcohol. Those 64% generally exhibited more eating disorder symptoms compared to the 36% who did not engage in these compensatory behaviours. However, issue with the study is the lack of testing/reporting as to whether any of the sample had any previous clinical diagnosis of eating disorders, therefore, making it impossible to distinguish whether those that did restrict caloric intake did so solely in response to alcohol or due to an eating disorder.

Despite Drunkorexia currently not being classified as an eating disorder and thus there not being a clinical diagnostic scale available, the Compensatory Eating and Behaviours in Response to Alcohol Consumption scale (CEBRACs) [20] has been developed to measure the amount and frequency with which participants exhibit ‘Drunkorexic’ behaviours. It was developed based upon the qualitative research of Peralta et al. (11) and Burke et al. [21]. When compared to the three subscales (drive for thinness, bulimia and body dissatisfaction) of the EDI-2 [22], the CEBRACs was found to be a valid measure [20, 23] which is now commonly used in drunkorexia studies [24, 25]. The CEBRACS scale is split into three time periods for participants to report any changes in their behaviour before drinking while under the effects of alcohol (during drinking) and after the effects of alcohol have worn off (after drinking). However, the CEBRACs is not used in clinical practice due to the lack of classification as a recognised clinical disorder – despite many arguing that Drunkorexia should be viewed as a clinical eating disorder.

Furthering the debate as to whether Drunkorexia should be classes as an eating disorder or substance use disorder, Choquette et al. [17], proposed that an adaptation of Fairburn’s [26] well-known transdiagnostic model of eating disorders, to explain the onset and maintenance of ‘Drunkorexic’ behaviours. The authors argued that Drunkorexia should be viewed as a food and alcohol disturbance, rather than as a disorder (either eating disorder or substance use disorder). In this adaptation, caloric restriction is engaging in as planned mechanism to either compensate for the caloric content of the alcohol they are consuming for appearance purposes, or to feel the intoxicating effects of alcohol stronger and quicker. Therefore, guilt and negative affect may occur if individuals do not restrict their eating prior to an episode of binge drinking, creating a problematic cycle prompted by overvaluation of weight and shape as restriction after drinking may occur. Fairburn’s [26] also included the idea of situational circumstances (such as life events and mood changes) which may be relevant to both restricting for caloric consumption and increasing the intoxication effects of alcohol. This adaptation and proposed name change incorporate all aspects of Drunkorexia while not limiting the behaviours as either an eating disorder or a substance abuse disorder. I might, therefore, serve as guidance for classification of Drunkorexia.

While an increasing number of studies investigating Drunkorexia are being conducted, there are still a plethora of unanswered questions, often as a result of methodological limitations [17]:

Firstly, studies are almost exclusively conducted with samples recruited from college (US) /university populations; possibly leading to the inaccurate assumption that Drunkorexia is only associated with university students [27]. Only one study, conducted by Lupi et al. [28] included both students (73%) and non-students (27%). The findings revealed Drunkorexia to be a common behaviour across their sample of 18–26-year-old Italian young adults and showed a significant correlation between these behaviours and both binge drinking and cocaine use. While it is a positive that the study was one of the first to include non-students, further research with a more even distribution of students and non-students is needed.

Secondly, most studies investigating Drunkorexia are conducted in the USA, Australia, and Canada [19, 27, 29]. Research from the UK is lacking. Only Scott et al. [30] recently (2020) published a qualitative study, where female interviewees were reported to have expressed calorie concerns with their alcohol use, while males were more likely to mention using the gym or exercising more before or after drinking. However, this study did not quantitively assess the prevalence and extent of Drunkorexia behaviours; thus, the extent to which these behaviours were engaged in, is unknown.

Thirdly, research investigating reasoning and motivations behind engagement in Drunkorexia is currently lacking. Possible predictors are thought to be emotional dysregulation, motivation to decrease negative mood [31, 32] and to prevent weight gain due to the calories of alcohol [20, 25]. Furthermore, Hill and Lego [29] investigated the relationship between Drunkorexia (i.e., the amount and frequency of Drunkorexia behaviours via the CEBRACS), body esteem and sensation-seeking with a sample of 448 undergraduate students. Body esteem is the dimension of self-esteem that primarily focuses on an individual’s perception of and attitude towards their weight and appearance [33]. Problems with body-/self-esteem are well reported for patients with eating disorders (mainly anorexia and bulimia nervosa) [34, 35], while increased sensation-seeking has been found to be a predictor of increased alcohol consumption, especially in university students [36]. It was found that poor body esteem and higher sensation seeking were significant predictors of ‘Drunkorexic’ behaviours. Body esteem was found to be a significant predictor of Drunkorexia, with the subscales appearance esteem and weight esteem being significant predictors of restriction, exercise, and dietary restraint. Sensation seeking was also a significant predictor of both drunkorexia and, unsurprisingly, alcohol use. To date, Drunkorexia is primarily seen as an eating disturbance, this study further highlights the disagreement as to whether it should be viewed as such or as a substance use issue [25]; or as both. Further investigating which role these predictors play in Drunkorexia will also yield insight into a better classification for Drunkorexia. While this study is the first to include the predictors of body esteem and sensation seeking and provides a good basis for further research, it is extremely limited in terms of generalisability, as all participants were undergraduate students from one introductory psychology course. It is also unclear as to whether participants were screened for any prior eating disorder diagnoses. This causes an issue for the findings, as it is a possibility that they might be reporting behaviours related to their diagnosis, but it is been interpreted as *drunkorexic* behaviours.

## The current study

The aim of the current study was to investigate the prevalence of compensatory behaviours (caloric restriction, increased exercise and bulimic tendencies) in response to alcohol consumption (also known as Drunkorexia) in students, non-students and previous students, as well as to understand the presence of possible predictors of these behaviours (body esteem and sensation seeking; as in Hill and Lego (29) study).

Firstly, it was predicted that all three groups of participants will engage similarly in Drunkorexia behaviours; and thus, that there will be no significant difference in Drunkorexia behaviours between students and non-students. This would provide evidence against the idea that *Drunkorexia* is only associated with the typical ‘university student lifestyle’ but much further spread than previously thought.

Secondly, low body esteem and high sensation seeking behaviours were explored as predictors of Drunkorexia; it was predicted that each would be a significant predictor for engagement in Drunkorexia behaviours for the whole sample (as in Hill & Lego).

## Methods

**Ethical approval** was granted by the University of Huddersfield; Department of Psychology Ethical Review Procedure. This study was conducted in line with the Ethics code of the British Psychological Society.

### Design

A between groups design was used for this study; there were three participant groups (students, not students and previous students) who all took part in the same questionnaires.

### Participants

Individuals aged 18–26, who are either students or non-students (including previous students) were eligible to participate. As aforementioned, the majority of participants in Drunkorexia research are students, leading to the assumption that the behaviours are only present in students. Lupi et al. [28], one of the only studies to include non-students, also used the age group of 18–26 years. Due to 18–26 years being the most common age group of university students [37]; this was chosen as the appropriate participant age range. Notably, Hill and Lego [29] did not have any limit on participants’ ages. However, their sample was limited to students, meaning that the need to control for age may not have appeared as pertinent in their study. Furthermore, individuals with previous diagnoses of eating disorders were also excluded. This means that a considerable confounding variable (presence of eating disorders) will have been removed. Individuals with previous diagnoses of alcohol use disorders were not excluded, as per comparable research in ‘*Drunkorexia’* (18,19,29)*.* This decision was based on the recurrent finding that many university students (thus, one of the subsamples in the study) see binge drinking, or excessive alcohol use, as central to their university experience, rather than a problem (38).

## Measures

### Demographics

A demographics questionnaire was developed by the authors to collect demographic information about participants; this included gender, age, occupation, how many days they have drank alcohol in the past month, weight and height. Inclusion of height and weight allowed the manual calculation of Body Mass Index (BMI).

### Drunkorexia (the compensatory eating and behaviours in response to alcohol consumption scale)

“*Drunkorexic*” behaviours were measured using the Compensatory Eating and Behaviours in Response to Alcohol Consumption Scale [20, 23]. The CEBRACS measures Drunkorexia behaviours across four factors (*alcohol effects, bulimia, diet and exercise*; and *restriction*) as well as a total score. Higher total scores indicate increased problems with compensatory behaviours, with the minimum score being 21 and the maximum score being 105 [20]. Internal consistency values for the sub-scales of the CEBRACs were as follows: *alcohol effects* α = 0.93; *bulimia* α = 0.35, *diet and exercise* α = 0.86 and *restriction* α = 0.69. The overall internal consistency of the CEBRACs was found to be good with α = 0.89. Only the *total* CEBRACS score was used in this study.

### Body-esteem (Body esteem scale)

The Body Esteem Scale for Adolescents and Adults was used to measure body-esteem (BESAA) [33]; it is a 23-item questionnaire that assesses body esteem across three subscales; *appearance esteem, weight esteem* and *attribution*. Higher scores on this measure indicate better overall body esteem. Internal consistency values for the current study were as follows: *appearance esteem* α = 0.923, *weight esteem* α = 0.925 and *attribution* α = 0.761. The internal consistency reliability of the overall BESAA was found to be extremely good; α = 0.948.

Hill and Lego [29] found that the *attribution* sub-scale of their sample had low internal consistency (α = 0.424) but good reliability for *appearance esteem* and *weight esteem* (AE: α = 0.825; WE: α = 0.825), thus only using *appearance* and *weight esteem* as predictors. However, the current study used all three sub-scales to understand all aspects of body esteem as a predictor of Drunkorexia.

### Sensation-seeking (brief sensation seeking scale)

The Brief Sensation Seeking Scale (BSSS) [38] was used to measure Sensation-seeking; it assesses sensation seeking across four domains (*thrill and adventure seeking*, *experience seeking, disinhibition* and *boredom susceptibility*), as well as a total score. The BSSS consists of eight items, with two items per subscale, measured on five-point Likert scales (strongly disagree—strongly agree). A higher total score indicates that participants either engage, or wish to engage, in riskier or thrilling activities. The BSS has been found to be a reliable and valid predictor of drug and alcohol use [38]. For the BSSS, Hill and Lego [29] reported adequate reliability with α = 0.737. In the current study, the BSSS also showed adequate overall reliability (α = 0.713); internal consistency reliability of the subscales varied (*thrill and adventure seeking* α = 0.495, *experience seeking* α = 0.184, *disinhibition* α = 0.608 and *boredom susceptibility* α = 0.265).

### Procedure

The survey was created on Qualtrics and advertised on campus, as well as shared across multiple social media sites. The study was also on the university’s research participation system, for students to complete in return for course credit. The order participants were presented with the surveys are as follows: information sheet, consent form, demographic questions, CEBRACS, BEESA, BSSS and the debrief sheet (Supplementary material 1).

### Analysis

Analyses were conducted on IBM SPSS *ver.* 26. After participants were removed (for either: incomplete answers, age, did not drink or explicit answers); negative items on the BESAA [33] were reversed scored. As it was assumed that any missing data at this point was missing at random, it was decided to do mean substitution for missing values; this allows analysis to still be completed on the dataset [39] and the creating of total scores of the scales, which is essential for the current study. The total scores for the CEBRACs, BESAA and BSSS were calculated, as well as the appropriate sub-scales.

## Results

### Demographics

#### Sample size

Initially, 170 participants accessed the survey link. Out of those, two did not consent (*n* = 2), 53 were removed due to not meeting the inclusion criteria of age (in detail: not being aged 18–25 (*n* = 17) or did not drink alcohol (*n* = 36)). Twenty participants dropped out prematurely (*n* = 20); thus, leaving a final sample size of 95. In detail, the sample consisted of ninety-five participants, twelve males and eighty-two females (mean age = 21.39, SD = 2.46, *n* = 94; one participant did not report their gender and age but was retained).

#### Age by occupation

The occupational breakdown of participants showed there were sixty-four students (*n* = 64, mean age = 20.55, SD = 2.02), eleven non-students (*n* = 11, mean age = 21.55, SD = 2.46) and twenty previous students who are now working (*n* = 19, mean age = 24.16, SD = 1.71). The overall sample mean age was 21.39 (SD = 2.46; *n* = 91; missing *n* = 1).

#### Gender and age

Out of the ninety-five participants, twelve identified as males and eighty-two females (mean age = 21.39, SD = 2.46, n = 94; missing *n* = 1, see above).

#### Alcohol consumption

The mean number of days that participants consumed alcohol (in relation to 30 days prior to the survey) was 6 days (SD = 4.77, *n* = 95, Range = 1–23). Using an independent samples *t*-test, no significant difference in days consuming alcohol between men (*n* = 12, *m* = 6.42, *SD* = 6.47) and women (*n* = 82*, m* = 5.95, *SD* = 4.55) were found; *t*(92) = 0.312, *p* = 0.756. A one-way ANOVA was conducted to investigate differences in alcohol use between the three occupation groups; no significant difference in alcohol use was found across the groups (*F* [2, 92] = 0.098, *p* = 0.907). These results suggest that there are no differences in days alcohol was consumed between the two genders, and occupations.

#### BMI

The mean for Body Mass Index (BMI) for men was *m* = 24.51 (*n* = 12, SD = 5.65) and for women *m* = 23.99 (*n* = 67, SD = 5.19).

#### CEBRACs

Reported *Drunkorexia behaviours* for men was *m* = 26.92 (*n* = 12, SD = 6.92) and for women; *m* = 33.52 (*n* = 82, SD = 11.63); there were no significant differences between the groups (Independent samples -test: *t*(92) = − 1.914, *p* = 0.059) (see Table 1). In terms of CEBRACs scores and occupation, previous students had a mean score of *m* = 34.60 (*n* = 20, SD = 11.01), non-students had a mean score of *m* = 31.18 (*n* = 11, SD = 14.06) and students had a mean score of *m* = 32.23 (*n* = 64, SD = 10.97). Further One-way ANOVAs were conducted to assess gender differences on the subscales of the CEBRACs, the ANOVAs were not significant (*p* > 0.05; range *p*: 0.081–0.597); suggesting no differences between groups on the subscales either.

**Table 1:** Descriptive statistics for gender

Variables	Total				Men				Women			
	*N*	*M*	SD	Range	*N*	*M*	SD	Range	*N*	*M*	SD	Range
BMI	80	24.04	5.20	14.98–42.32	12	24.51	5.65	16.72–36.38	67	23.99	5.19	14.98–42.32
CEBRACs total score	95	32.61	11.29	21–74	12	26.92	6.92	21–42	82	33.52	11.63	21–74
Body esteem total score	93	62.62	17.53	26–102	12	74.08	13.81	55–96	80	60.86	17.55	26–102
Sensation seeking total score	90	24.90	5.35	13–38	12	25.33	6.33	14.37	77	24.78	5.24	13–38

There is no significant difference in BMI between genders

### Testing of hypothesis 1; Drunkorexia behaviours across groups

As predicted, participants in all three groups (students, non-students and previous students) engaged in *Drunkorexia behaviours*, as reflected by their CEBRACs total score (mean CEBRACs scores range: 31.2–34.6; Table 2). A one-way ANOVA was conducted to investigate the differences between the groups and found no significant differences on engagement in *Drunkorexia behaviours* between the three groups. (*F* [2, 92] = 0.429, *p* = 0.652). For completion, two further One-way ANOVAs were conducted to investigate differences in *body-esteem* (*F* [2, 90] = 1.384, *p* = 0.256) and *sensation seeking* (*F* [2, 87] = 1.127, *p* = 0.329) between the three groups; both were non-significant.

**Table 2:** Descriptive statistics for occupation groups

Variables	Total				Non-student				Student				Previous student			
	*N*	*M*	SD	Range	*N*	*M*	SD	Range	*N*	*M*	SD	Range	*N*	*M*	SD	Range
BMI	80	24.04	5.20	14.98–42.32	8	23.92	5.71	18.15–37.20	54	23.64	4.81	14.98–36.98	18	25.29	6.16	17.64–42.32
CEBRACs: total	95	32.61	11.29	21–74	11	31.18	14.06	21–61	64	32.23	10.97	21–74	20	34.60	11.01	23–60
CEBRACs: alcohol effects	95	12.62	6.70	7–35	11	11.73	7.56	7–29	64	12.77	6.58	7–35	20	12.65	6.93	7–35
CEBRACs: bulimia	95	6.49	1.16	6–12	11	6.36	0.67	6–8	64	6.52	1.23	6–12	20	6.50	1.15	6–10
CEBRACs: exercise	95	10.65	5.20	6–28	11	10.09	5.39	6–19	64	10.16	4.67	6–26	20	12.55	6.44	6–28
CEBRACs: restriction	95	2.84	1.67	2–10	11	3.00	1.55	2–6	64	2.80	1.80	2–10	20	2.90	1.29	2–6
BEESA Total Score	93	62.62	17.53	26–102	11	61.00	19.69	34–96	62	64.62	18.12	26–102	20	57.30	13.69	31–84
BEESA: appearance	93	27.37	8.49	10–48	11	27.91	10.50	12–48	62	27.85	8.65	10–44	20	25.55	6.82	14–38
BEESA: Weight Esteem	93	21.08	7.73	8–38	11	20.36	9.47	8–36	62	22.18	7.80	8–38	20	18.05	5.74	8–30
BEESA: attribution	93	14.18	3.59	6–25	11	12.73	2.83	6–16	62	14.59	3.78	7–25	20	13.70	3.23	9–21
BSS: Total	90	24.90	5.35	13–38	11	22.64	4.57	14–29	61	25.20	5.08	13–37	18	25.28	6.54	17–38
BSS: thrill and adventure seeking	90	5.72	2.00	2–10	11	5.36	1.80	3–8	61	5.84	1.99	2–10	18	5.56	2.23	2–10
BSS experience seeking	90	6.66	1.71	2–10	11	6.18	1.54	4–8	61	6.61	1.66	2–10	18	7.11	1.97	4–10
BSS: disinhibition	90	5.81	1.97	2–10	11	4.91	1.87	2–7	61	6.02	2.01	2–10	18	5.67	1.78	2–7
BSS: boredom susceptibility	90	6.71	1.66	3–10	11	6.18	1.40	4–8	61	6.74	1.61	3–10	18	6.94	1.98	3–10

Further One-way ANOVAs were conducted to assess differences on the subscales of the CEBRACs between the three groups. The ANOVAs were not significant (*p* > 0.05; range *p*: 0.186–0.923); suggesting no differences between groups on the subscales either.

### Regressions

Firstly, a Multiple Regression Analysis (Enter Method) was conducted to investigate how well the variables *body esteem* t*otal* and *sensation seeking total* predicted the outcome variable *Drunkorexia* (via total CEBRACs) score. Both *body-esteem total score* (*t* = − 4.69, *p* ≤ 0.001) and *sensation-seeking total score* (*t* = 3.395, *p* ≤ 0.001) were found to be significant predictors of engagement in Drunkorexia behaviours (*F*(2, 87) = 19.91, *p* ≤ 0.001). Overall, they accounted for 30% of the variance of CEBRAC scores. The regression line shows ‘Drunkorexia behaviours (CEBRACs total) = 33.74 + (− 0.271 × Body Esteem Total) + (0.64 × Sensation Seeking Total). These results indicated that participants with higher sensation seeking scores and lower body esteem scores were more likely to have higher CEBRACs scores, thus, engage in more Drunkorexia behaviours.

Secondly, when conducting the same regression analysis with individual factors of the *BEESA scale* (Attribution, Weight Esteem and Appearance esteem) as well as *total BSSS* scores; only *Appearance Esteem* (*t* = −2.62; *p* = 0.011) and *BSSS total score* (*t* = 2.66; *p* = 0.009) predicted engagement in Drunkorexia behaviours (*F*(4, 85) = 12.14, *p* ≤ 0.000). *Weight esteem* (*t* = − 1.18, *p* = 0.241) as well as *attribution* (*t* = 1.67; *p* = 0.097) were not found to be a significant predictor of Drunkorexia behaviours. Overall, this model predicted 33% of the variance in scores. The regression line shows Drunkorexia behaviours (CEBRACs total) = 32.14 + (− 0.54 × appearance esteem) + (0.549 × attribution) + (− 0.249 × weight esteem) + (0.507 × sensation seeking).

Thirdly, when only including the significant predictors (*sensation seeking total, appearance esteem*) into the multiple regression; the following regression equation was calculated (*F*(2, 87) = 21.77, *p* < 0.001).; explaining 32% of the variance: Drunkorexia (Total CEBRACS total score) = 34.52—(0.594 × Appearance esteem) + (0.578 × Sensation seeking).

Fourthly, when conducting the multiple regression with individual factors of the BSSS (Thrill and adventure seeking, boredom susceptibility, disinhibition factor & experience seeking) as well as *appearance esteem*; *appearance esteem* (*t* = − 5.034, *p* ≤ 0.000) and Disinhibition (*t* = 2.031, *p* = 0.045) were found to be significant predictors of Drunkorexia (*F*(5, 84) = 8.83, *p* ≤ 0.000). Overall, this model predicted 31% of the variance of scores; the regression equation is as follows: Drunkorexia behaviours (CEBRACs total score) = 35.562 + (− 0.605 × appearance esteem) + (0.261 × experience seeking) + (0.403 × boredom susceptibility) + (0.399 × thrill and adventure seeking) + (1.193 × disinhibition).

Fifthly, when only conducting the multiple regression with the significant predictors (*appearance esteem* and *disinhibition),* the following regression equation was calculated (*F*(2, 87) = 21.47, *p* ≤ 0.000); explaining 32% of the variance: Drunkorexia (CEBRACs total score) = 41.48 + (1.506 × disinhibition) + (− 0.642 × appearance esteem).

## Discussion

The aim of the current study was to investigate the differences in Drunkorexia between students, non-students and previous students, as well as to understand the presence of possible predictors of these behaviours in the sample (body esteem, sensation seeking).

As hypothesised, there was no significant difference found between students, non-students, and previous students on engagement with *Drunkorexia* behaviours. This is a novel and important finding, as, to date, no other study has investigated, and reported, this finding. The current findings highlight that Drunkorexia is present in populations other than current students, which so far has been overlooked. Tackling Drunkorexia behaviour, via interventions in clinical and campus settings [16, 29], should, therefore, not be limited to student-only or clinical (eating disorder treatment) samples. It might, therefore, especially be a problem for the age group of 18–26 years; regardless of occupation. The study further highlights that that non-students should be included in future research investigating Drunkorexia, as they also engage in these behaviours (despite Drunkorexia typically being associated with university students).

Second, the regressions analysis showed both *low body esteem* and *high sensation seeking* were significant predictors of engagement with *Drunkorexia* behaviours. This replicates some of the findings by Hill and Lego [29]. However, upon further investigation, only *appearance esteem* and *disinhibitio*n were found to be the significant individual predictors of engagement with Drunkorexia behaviours (rather than total scale scores); explaining about 30% of the variance in scores.

This is in contrast to Hill and Lego [29] who found *weight esteem*, *appearance esteem* and *overall sensation seeking* the significant predictors of Drunkorexia. Hill and Lego (29) did not further explore the factors underlying the BSSS as predictors of Drunkorexia. Therefore, the current research extends knowledge on the predictors of Drunkorexia, especially within the sensation seeking aspect. Disinhibition, as a central concept to sensation seeking, refers to the likelihood of a person engaging in less acceptable forms of sensation seeking, such as reckless behaviours, poor decision making and a reduced sensitivity to future consequences [38, 40, 41]. It has also been linked to excessive alcohol consumption [42]; thus, it is not entirely surprising to find this a predictor of a behaviour that is associated with substance abuse.

Furthermore, while Hill and Lego [29] also found weight esteem to be a significant predictor for engagement in Drunkorexia behaviours, the current study did not find the predictor of weight esteem to be significant (unlike Hill & Lego). A potential reason for this might be the exclusion of people with a previous diagnoses of eating disorders; which was not an exclusion criterion in past research. Hill and Lego did also not have this inclusion. Therefore, in their sample of 488 students there is a high chance that participants with EDs were included; considering estimates that between 2 and 20% of higher education students, especially females, struggle with EDs, and that the age groups included in the current study sample (i.e., 18–26 years) include the highest risk group for the development of EDs [43, 44].

Thus, it could be suggested that Drunkorexia, for patients with EDs, may be a symptom of their illness (engagement in Drunkorexia with the intent of not gaining weight), whereas for people without EDs, engagement in Drunkorexia behaviours may be the result of wanting to feel the effects of alcohol quicker to increase their appearance esteem (and thus their confidence). It could, therefore, be further suggested that Drunkorexia, therefore, acts mainly as a compensatory behaviour to mask low appearance confidence in individuals with high disinhibition levels. These are important findings; as they link to the debate as to whether Drunkorexia is driven by similar motivations as eating disorders or substance abuse disorder [16, 17]. The current results now suggest that it could possibly be either a symptom of EDs, i.e., engagement in Drunkorexia to prevent weight gain from alcohol, while the disinhibition factors of increased sensation-seeking may explain the aspect of substance use as a compensatory behaviour to feel the effects of alcohol quicker (i.e., to feel better about their appearance). Scott et al. [30] showed through their qualitative study how young people often change their eating and drinking habits to match their social situation, possibly why people without eating disorders still participate in drunkorexia behaviours.

The result also provide further support for Choquette et al.’s [17]’ adapted model of Fairburn’s [26] transdiagnostic model of eating disorders; suggesting that Drunkorexia should be seen more as an eating disturbance in response to alcohol (or a Food and Alcohol Disturbance), rather than a clinical disorder.

Furthermore, women were found to have slightly higher CEBRACs scores than men, but this difference was found to be non-significant; this finding is in line with previous research [20, 29]. However, it has to be acknowledged that the gender split as uneven in the current study. Nonetheless, Hill and Lego [29] as well as Rahal et al. [20]’s studies showed women did have slightly higher CEBRACs scores than men, indicating women exhibited more Drunkorexia behaviours than men—but as above, the difference was non-significant. Eisenberg and Fitz [45] found that women reported significantly more Drunkorexia behaviour than men. However, in Eisenberg and Fitz [45]’s study, the CEBRACs was not used but a single-item measure regarding restricting food before drinking. Eisenberg and Fitz’s study, therefore, may not accurately represent behaviours that make up Drunkorexia (such as exercise, diet, and bulimic tendencies). Qualitative research has highlighted how women often report the idea of being aware of ‘empty calories’, and being concerned with the caloric content of their drinks more frequently than men (30).

## Limitations

A number of limitations of the present study warrant discussion.

Firstly, the main aim of the current study was to investigate the differences between students, non-students and previous students; however, the sample consisted of 67.4% students. Lupi et al. [28] is one of the only studies to have included non-students and their sample included 73.1% students. While the current study had a greater representation of non-students, the distribution between groups was not as even as would have been preferred.

Secondly, the bulimia subscale of the CRBRACS had low internal consistency; however, as the overall internal consistency reliability of the CEBRACs was high and the subscale was not used as either an individual predictor or outcome measure by itself, this limitation may have been overcome.

A third limitation is the use of self-report questionnaires. It is unclear whether the answers are true representations of the participants. It is known that people who exhibit disordered eating behaviours, often rationalise their behaviour as a healthy lifestyle choice [46]. This is likely due to the emphasis of the role of caloric imbalance in obesity and weight gain in the modern day, meaning vulnerable participants restrict calories to avoid this. Due to this, participants may not disclose the extent to which they participate in these behaviours as to not alert a problem as they cannot explain themselves further in a quantitative questionnaire. The use of self-report measures, however, did allow for a larger sample than would be possible with interviews or a narrative approach.

In addition, it is worth mentioning that the way in which alcohol consumption was reported in the demographic study may not be a true representation. By reporting how many days the participants drank alcohol, it may seem like a lower number would mean a smaller consumption, but someone that only drank for a few days, may have drank more units than someone who drank regularly. Hill and Lego [29] measured alcohol by asking participants to report the average amount of drinks they typically drank on a weekday and the weekend. While this still did not give an exact amount that participants drank, it allowed participants to give a more accurate representation in comparison to the current study. An alternative would be the Timeline Follow Back method for recording alcohol consumption (TLFB; Sobell & Sobell, 1992). This is a retrospective self-report measure that uses a calendar-based method for participants to report the days that they drank alcohol, as well as the number of standard drinks consumed on said days. From this a number of things can be generated; drinks per drinking day (DDD), percentage of heavy drinking days (PHDD) and percentage of day abstinent (PDA), allowing for a more accurate measure of the amount and frequency consumed [48].

### Recommendations for future work

Given that the current study, along with a number of other studies [27–29], were cross-sectional, future research should address if there are any changes in Drunkorexia behaviours longitudinally for any of the occupation groups. It is also important to consider that the current study collected date in the second term of the university year. It would be logical to assume that students may consume more alcohol in the first term of the university year (e.g., during ‘Freshers’, Christmas etc.) in comparison to the second term. This may have affected results, as it is thought that when alcohol consumption is higher, people exhibit more Drunkorexia behaviours [27]. In addition, research needs to be extended to investigate prevalence of Drunkorexia amongst older age groups; to potentially further inform targeting and intervention development. This, therefore, highlights the need for further longitudinal research of the current age groups.

The inclusion/exclusion criteria of this study, as well as past studies, also warrants recommendations for further research: while the current study excluded the participation of individuals with previous diagnoses of eating disorders, it did not exclude participants with previous diagnoses of substance use disorders. Thus, excluding participants with a history of alcohol/substance use issues is thus necessary for future research, to avoid behaviours associated with alcohol misuse being reported as ‘*Drunkorexia*’. This exclusion criterion has not been considered in past research.

In terms of recommendations for future interventions, Drunkorexia or any disordered eating behaviours in response to alcohol consumption should be taken seriously by both healthcare providers and universities. The severity of both, disordered eating and risky alcohol behaviours, should not be overlooked and adequate education on the subjects should be introduced at school-age to prevent these behaviours developing in adolescence and early adulthood.

## Conclusion

The present study indicates that both poor body esteem and increased sensation seeking, specifically appearance esteem and disinhibition, are predictors of Drunkorexia behaviour. It also provides further support for the classification of Drunkorexia: the results support Choquette et al.’s [17] argument that Drunkorexia should be viewed as an eating disturbance, as a result of alcohol (or a Food and Alcohol Disturbance), rather than a clinical disorder (eating disorder, substance use disorder). In addition, the results provide support that thus warrants immediate research and clinical attention due to the prevalence of Drunkorexia across groups, considering damaging nature of compensatory behaviours prior, or in response, to alcohol consumption.

In summary, the results suggest the need for further research into motivations behind these complex behaviours, as well as the need for interventions across all occupations, not just limiting them to students.

### What is already known about the subject?

Restricted eating in response to alcohol consumption has been labelled *Drunkorexia*. Prevalence in non-student samples & motivators behind Drunkorexia are currently unclear.

### What does this study add?

Students, previous students & non-students were equally as likely to engage in Drunkorexia. Low body esteem & high sensation seeking tendencies were significant predictors*.*

## Electronic supplementary material

Below is the link to the electronic supplementary material.

Supplementary file1 (DOCX 32 kb)

## Data Availability

The data are available upon request to the corresponding author. This study was conducted in accordance with the Ethics Code published by the British Psychological Society and approved by the University of Huddesfield’s Department of Psychology Ethical Review Committee (Dr Tim Gommersal and Dr Gurjog Bagri). Informed consent was obtained from participants, no deception took place. Participation was voluntary, anonymous and confidential. Participants were debriefed.

## References

[1] NHS digital. (2019). Statistics on Alcohol, England 2019 [PAS]—part 2. Statistics on alcohol.

[2] Welch KA (2017). Alcohol consumption and brain health. BMJ (Online).

[3] Public Health England. (2019). Local Alcohol Profiles for England. Retrieved May 10, 2020, from https://fingertips.phe.org.uk/profile/local-alcohol-profiles/data#page/6/gid/1938132833/pat/6/par/E12000006/ati/102/are/E10000015/cid/4

[4] Loose TT, Acier D, Andretta JR, Cole JC, McKay MT, Wagner V, Worrell FC (2018). Time perspective and alcohol-use indicators in France and the United Kingdom: results across adolescents, university students, and treatment outpatients. Addiction Res Theory.

[5] Wilkinson B, Ivsins A (2017). Animal house: University risk environments and the regulation of students’ alcohol use. Int J Drug Policy.

[6] Blank ML, Connor J, Gray A, Tustin K (2016). Alcohol use, mental well-being, self-esteem and general self-efficacy among final-year university students. Soc Psychiatry Psychiatr Epidemiol.

[7] Maylor EA, Rabbitt PMA, Sahgal A, Wright C (1987). Effects of alcohol on speed and accuracy in choice reaction time and visual search. Acta Physiol (Oxf).

[8] Oscar-Berman M, Marinković K (2007). Alcohol: Effects on neurobehavioral functions and the brain. Neuropsychol Rev.

[9] Thoma P, Friedmann C, Suchan B (2013). Empathy and social problem solving in alcohol dependence, mood disorders and selected personality disorders. Neurosci Biobehav Rev.

[10] Hahn RG, Norberg Å, Jones AW (1997). “Overshoot” of ethanol in the blood after drinking on an empty stomach. Alcohol Alcohol.

[11] Peralta RL (2002). Alcohol use and the fear of weight gain in college: Reconciling two social norms. Gender Issues.

[12] Kelly-Weeder S (2011). Binge drinking and disordered eating in college students. J Am Acad Nurse Practitioners.

[13] Cummings JR, Tomiyama AJ (2018). Bidirectional associations between eating and alcohol use during restricted intake. Curr Addiction Rep.

[14] Bryant JB, Darkes J, Rahal C (2012). College students compensatory eating and behaviors in response to alcohol consumption. J Am Coll Health.

[15] Kershaw, S. (2008). Starving themselves, cocktail in hand. The New York Times.

[16] Hunt TK, Forbush KT (2016). Is “drunkorexia” an eating disorder, substance use disorder, or both?. Eat Behav.

[17] Choquette EM, Rancourt D, Kevin Thompson J (2018). From fad to FAD: A theoretical formulation and proposed name change for “drunkorexia” to food and alcohol disturbance (FAD). Int J Eat Disord.

[18] Roosen KM, Mills JS (2015). Exploring the motives and mental health correlates of intentional food restriction prior to alcohol use in university students. J Health Psychol.

[19] Knight A, Castelnuovo G, Pietrabissa G, Manzoni GM, Simpson S (2017). Drunkorexia: an empirical investigation among Australian female university students. Aust Psychol.

[20] Rahal CJ, Bryant JB, Darkes J, Menzel JE, Thompson JK (2012). Development and validation of the compensatory eating and behaviors in response to alcohol consumption scale (CEBRACS). Eat Behav.

[21] Burke SC, Cremeens J, Vail-Smith K, Woolsey C (2010) Drunkorexia: calorie restriction prior to alcohol consumption among college freshman. J Alcohol Drug Educ.

[22] Garner DM (1991). Eating disorder inventory 2: professional manual. Int J Eat Disorder.

[23] Rahal CJ, Bryant JB, Darkes J, Menzel JE, Thompson JK (2012). Compensatory eating and behaviors in response to alcohol consumption scale. PsycTESTS.

[24] Peralta RL, Barr PB (2017). Gender orientation and alcohol-related weight control behavior among male and female college students. J Am Coll Health.

[25] Pompili S, Laghi F (2018). Drunkorexia: Disordered eating behaviors and risky alcohol consumption among adolescents. J Health Psychol.

[26] Fairburn, C. G. (2008). Eating disorders: The transdiagnostic view and the cognitive behavioral theory. Cognitive Behavior Therapy and Eating Disorders.

[27] Barry AE, Piazza-Gardner AK (2012). Drunkorexia: Understanding the co-occurrence of alcohol consumption and eating/exercise weight management behaviors. J Am Coll Health.

[28] Lupi M, Martinotti G, Di Giannantonio M (2017). Drunkorexia: an emerging trend in young adults. Eating Weight Dis.

[29] Hill EM, Lego JE (2019). Examining the role of body esteem and sensation seeking in drunkorexia behaviors. Eating Weight Dis.

[30] Scott S, Muir C, Stead M, Fitzgerald N, Kaner E, Bradley J, Adamson A (2020). Exploring the links between unhealthy eating behaviour and heavy alcohol use in the social, emotional and cultural lives of young adults (aged 18–25): A qualitative research study. Appetite.

[31] Ward RM, Galante M (2015). Development and initial validation of the Drunkorexia Motives and Behaviors scales. Eat Behav.

[32] Laghi F, Pompili S, Bianchi D, Lonigro A, Baiocco R (2019). Psychological characteristics and eating attitudes in adolescents with drunkorexia behavior: an exploratory study. Eating Weight Dis.

[33] Mendelson BK, Mendelson MJ, White DR (2001). Body-esteem scale for adolescents and adults. J Pers Assess.

[34] Mendelson BK, McLaren L, Gauvin L, Steiger H (2002). The relationship of self-esteem and body esteem in women with and without eating disorders. Int J Eat Disord.

[35] Vinkers CDW, Evers C, Adriaanse MA, de Ridder DTD (2012). Body esteem and eating disorder symptomatology: the mediating role of appearance-motivated exercise in a non-clinical adult female sample. Eat Behav.

[36] Yanovitzky I (2006). Sensation seeking and alcohol use by college students: Examining multiple pathways of effects. J Health Commun.

[37] HESA Higher Education Student Statistics. (2020). Higher Education Student Statistics: UK, 2017/18—Student numbers and characteristics. Higher Education Student Statistics: UK, 2017/18—Student numbers and characteristics* | HESA*. Retrieved May 10, 2020, from https://www.hesa.ac.uk/news/17-01-2019/sb252-higher-education-student-statistics/numbers

[38] Hoyle RH, Stephenson MT, Palmgreen P, Lorch EP, Donohew RL (2002). Reliability and validity of a brief measure of sensation seeking. Personality Individ Differ.

[39] Kang H (2013). The prevention and handling of the missing data. Korean J Anesthesiol.

[40] Orlebeke JF, Van Der Molen MW, Dolan C, Stoffels EJ (1990). The additive factor logic applied to the personality trait disinhibition. Personality Individ Differ.

[41] Crone EA, Vendel I, van der Molen MW (2003). Decision-making in disinhibited adolescents and adults: Insensitivity to future consequences or driven by immediate reward?. Personality Individ Differ.

[42] Carlson SR, Johnson SC, Jacobs PC (2010). Disinhibited characteristics and binge drinking among university student drinkers. Addict Behav.

[43] Kugu N, Akyuz G, Dogan O, Ersan E, Izgic F (2006). The prevalence of eating disorders among university students and the relationship with some individual characteristics. Aust N Z J Psychiatry.

[44] Tavolacci MP, Grigioni S, Richard L, Meyrignac G, Déchelotte P, Ladner J (2015). Eating disorders and associated health risks among university students. J Nutr Educ Behav.

[45] Eisenberg MH, Fitz CC (2014). “Drunkorexia”: Exploring the who and why of a disturbing trend in college students’ eating and drinking behaviors. J Am Coll Health.

[46] Musolino C, Warin M, Wade T, Gilchrist P (2015). “Healthy anorexia”: The complexity of care in disordered eating. Soc Sci Med.

[47] Sobell LC, Sobell MB, Litten RZ, Allen JP (1992). Timeline Follow-Back. Measuring alcohol consumption.

[48] Dulin, P. L., Alvarado, C. E., Fitterling, J. M., & Gonzalez, V. M. (2017). Comparisons of alcohol consumption by timeline follow back vs.smartphone-based daily interviews. Addiction Res Theory. https://doi.org/10.1080/16066359.2016.1239081 10.1080/16066359.2016.1239081 PMC5695707 29170622

