# Supplementary material for: Drunkorexia: is it really “just” a university lifestyle choice?
Source: Eat Weight Disord. 2020 Oct 30;26(6):2021–31. doi: 10.1007/s40519-020-01051-x (PMC8292268; doi:10.1007/s40519-020-01051-x)
Supplement: Supplementary file 1 — Supplementary file1 (DOCX 32 kb) [file 40519_2020_1051_MOESM1_ESM.docx]

Drunkorexia

Survey Flow

Block: Information Sheet (1 Question)

Standard: Consent Form (2 Questions)

Standard: Demographic Information (9 Questions)

Standard: CEBRACS (4 Questions)

Standard: BES (1 Question)

Standard: BSS (1 Question)

Standard: Debrief (1 Question)

| Page Break |  |
| --- | --- |

Start of Block: Information Sheet

Q1 ***Dear participant,***   We would like to invite you to take part in our research study. Before you decide, we would like you to understand why the research is being done and what it would involve for you. If you have any questions about the information provided, please contact *Bethany Griffin at u1755073@unimail.hud.ac.uk* or their supervisor *Katharina Vogt at K.Vogt@hud.ac.uk*.   **What is the purpose of this study?** The current study aims to explore the link between diet choices and alcohol consumption in both students and non-students.   **Can I take part?** The study requires both current students and non-students, aged between 18 and 26 with no prior clinical diagnosis of an eating disorder.   **What will the study involve?** You will be asked to complete 4 questionnaires: a demographic questionnaire, a questionnaire focusing on behaviours relating to drinking, a body-esteem questionnaire and a questionnaire regarding the personality trait of sensation seeking.   **Are there any risks or benefits of taking part?** It has to be stated that for some people, reflecting on eating behaviours and alcohol consumption can be stressful. We ask that if you are concerned about your participation that you don’t participate for your own wellbeing. By taking part in the study, you will be providing data for a topic that hasn’t really been looked into in both a UK and non-student population. Huddersfield University students will be provided with SONA credits in return for their participation.   **Do I have to take part and will my involvement with the study be kept confidential?** Your participation in the study is completely voluntary and can withdraw from the study at any time, right up until you submit the survey. Once you have submitted the survey, you won’t be able to withdraw. This is due to the data been collated immediately after submission. However, your data will be completely anonymised, and you won’t be able to be identified at any stage of both analysis and the write-up process.   **What will happen to the results of the research study?** The results from the study will be written up for my dissertation and then presented after submission. The data will remain completely anonymous and won’t be able to be traced back to participants. The data will be stored at the university for an indefinite amount on a password-protected computer; the data may lead to publication in appropriate journals. However, no individual will be identified as having taken part in this research.

End of Block: Information Sheet

Start of Block: Consent Form

Q2
 **1.** I confirm that I have read and understand the information sheet for the above study and have had the opportunity to ask questions using the researcher contact details. 
 **2.** I understand that my participation is voluntary and that I am free to withdraw at any time before submitting the survey.
 **3.** I understand that once I have submitted the questionnaire, I cannot withdraw my answers, but I know that the data will be completely anonymous and won’t be able to be identified during analysis or the report. 
 **4.** I understand that data collected in the study may be looked at by the research group. I give permission for these individuals to have access to these records and to collect, store, analyse and publish information obtained from my participation in this study.
 **5.** I understand that my personal details will be kept confidential. I understand that the questionnaire may include sensitive information regarding my eating and drinking habits. I agree to take part in the above study.
  

Q3 **By continuing, you understand that you can not withdraw your answers once the survey has been submitted.**

- Yes, I consent. (1)
- No, I do not wish to take part in the study. (2)

Skip To: End of Survey If By continuing, you understand that you can not withdraw your answers once the survey has been sub... = No, I do not wish to take part in the study.

End of Block: Consent Form

Start of Block: Demographic Information

Q4 **What gender do you identify as?**

- Male (1)
- Female (2)
- Other (3) ________________________________________________

Q5 **What is your age?**

________________________________________________________________

Skip To: End of Survey If Condition: What is your age? Is Greater Than 26. Skip To: End of Survey.

Skip To: End of Survey If Condition: What is your age? Is Less Than 18. Skip To: End of Survey.

Q20 ***Please answer only if you feel comfortable to do so:***
**What is your height? Please indicate if you have used cm or feet.**
 *e.g. 155cm or 5'1 ft*

________________________________________________________________

Q21
***Please answer only if you feel comfortable to d****o so:*
What is your weight? Pl**ease indicate if you have used stone or kg.** *e.g. 11 st 0 lbs or 70 kg*

________________________________________________________________

Q7 **What is your occupation?**

- Student (1)
- Non-student (2)
- Non-student but has previously attended university (3)

Display This Question:

If What is your occupation? = Student

Q8 **Which course are you currently enrolled to?**

________________________________________________________________

Display This Question:

If What is your occupation? = Non-student

Q9 **What is your current job title?**

________________________________________________________________

Display This Question:

If What is your occupation? = Non-student but has previously attended university

Q10 **What is your current job title and your previous degree subject?**

________________________________________________________________

Q11 **Over the past 30 days, roughly how many of those have you consumed alcohol?**

________________________________________________________________

Skip To: End of Survey If Condition: Over the past 30, roughly h... Is Equal to 0. Skip To: End of Survey.

End of Block: Demographic Information

Start of Block: CEBRACS

Q13
**Compensatory Eating Behaviours Related to Alcohol Consumption Scale.**
**Instructions:**
 Please read each of the following statements very carefully and respond accurately and honestly. All of these statements reflect actual behaviors you may have done in the past 3 months. You will be asked whether you have done any of the behaviors before, during, or after drinking alcohol. Please read carefully because many of the statements are closely related to each other. Drinking refers to drinking any alcoholic beverages such as: beer, wine, wine coolers or spirits, hard liquors or mixed drinks.

Q14 **Before Drinking:**For the following statements think about behaviors you engaged in **before** you anticipated drinking alcohol, referring back to the last 3 months.

|  | Never (1) | Rarely (2) | Sometimes (3) | Often (4) | Always (5) |
| --- | --- | --- | --- | --- | --- |
| 1) I have eaten less than usual during one or more meals before drinking to get drunker. (1) |  |  |  |  |  |
| 2) I have exercised before drinking to make up for the calories in alcohol that I anticipated consuming. (2) |  |  |  |  |  |
| 3) I have eaten less than usual during one or more meals before drinking to feel the effects of alchol faster. (3) |  |  |  |  |  |
| 4) I have skipped one or more meals before drinking to make up for the number of calories in alcohol that I anticipated consuming. (4) |  |  |  |  |  |
| 5) I have taken laxatives before drinking to make up for the calories in alcohol that I anticipated consuming. (5) |  |  |  |  |  |
| 6) I have skipped one or more meals before drinking to feel the effects of alcohol faster. (6) |  |  |  |  |  |

Q15 **While Under the Effects of Alcohol:**For the following statements, think about behaviours you engaged in **while** you were drinking or under the effects of alcohol, referring back to the last 3 months.

|  | Never (1) | Rarely (2) | Sometimes (3) | Often (4) | Always (5) |
| --- | --- | --- | --- | --- | --- |
| 7) I have eaten less than usual while I was drinking because I wanted to feel the effects of the alcohol faster. (1) |  |  |  |  |  |
| 8) I have taken diuretics while I was drinking to make up for the calories in alcohol that I was consuming. (2) |  |  |  |  |  |
| 9) I have not eaten at all while I was drinking because I wanted to feel the effects of the alcohol faster. (3) |  |  |  |  |  |
| 10) I have eaten low-calorie or low-fat foods while I was drinking to make up for the calories in alcohol that I was consuming. (4) |  |  |  |  |  |
| 11) I drank low-calorie beer or alcoholic drinks to get fewer of the calories that are in alcohol. (5) |  |  |  |  |  |
| 12) I have eaten less than usual while I was drinking because I wanted to get drunker. (6) |  |  |  |  |  |
| 13) I have taken laxatives while I was drinking to make up for the calories in alcohol that I was consuming. (7) |  |  |  |  |  |
| 14) I have not eaten at all while I was drinking because I wanted to get drunker. (8) |  |  |  |  |  |

Q14 **After Drinking:**For each of the following statements, think about behaviors you have engaged in**after** you had been drinking alcohol and were no longer under the effects of alcohol, referring back to the last 3 months.

|  | Never (1) | Rarely (2) | Sometimes (3) | Often (4) | Always (5) |
| --- | --- | --- | --- | --- | --- |
| 15) I have taken diuretics to make up for the calories in alcohol that I had consumed previously while I was under the effects of alcohol. (1) |  |  |  |  |  |
| 16) I have eaten low-calorie or low-fat foods during one or more meals to make up for the calories in alcohol that I had consumed previously while I was under the effects of alcohol. (2) |  |  |  |  |  |
| 17) I have taken laxatives to make up for the calories in alcohol that I had consumed previously while I was under the effects of alcohol. (3) |  |  |  |  |  |
| 18) I have exercised to make up for the calories in alcohol that I had consumed previously while I was under the effects of alcohol.   (4) |  |  |  |  |  |
| 19) I have made myself vomit to make up for the calories in alcohol that I had consumed previously while I was under the effects of alcohol.   (5) |  |  |  |  |  |
| 20) I have eaten less than usual during one or more meals to make up for the calories in alcohol that I had consumed previously while I was under the effects of alcohol. (6) |  |  |  |  |  |
| 21) I have skipped an entire day or more of eating to make up for the calories in alcohol that I had consumed previously while I was under the effects of alcohol. (7) |  |  |  |  |  |

End of Block: CEBRACS

Start of Block: BES

Q16
**Body-Esteem Scale for Adolescents and Adults.**
**Instructions:**
 Indicate how often you agree with the following statements ranging from "never" to "always".

|  | Never (1) | Rarely (2) | Sometimes (3) | Often (4) | Always (5) |
| --- | --- | --- | --- | --- | --- |
| 1. I like what I look like in pictures. (1) |  |  |  |  |  |
| 2. Other people consider me good looking. (2) |  |  |  |  |  |
| 3. I'm proud of my body. (3) |  |  |  |  |  |
| 4. I am preoccupied with trying to change my body weight. (4) |  |  |  |  |  |
| 5. I think my appearance would help me get a job. (5) |  |  |  |  |  |
| 6. I like what I see when I look in the mirror. (6) |  |  |  |  |  |
| 7. There are lots of things I'd like to change about my looks if I could. (7) |  |  |  |  |  |
| 8. I am satisfied with my weight. (8) |  |  |  |  |  |
| 9. I wish I looked better. (9) |  |  |  |  |  |
| 10. I really like what I weigh. (10) |  |  |  |  |  |
| 11. I really wish I looked like someone else. (11) |  |  |  |  |  |
| 12. People my own age like my looks. (12) |  |  |  |  |  |
| 13. My looks upset me. (13) |  |  |  |  |  |
| 14. I'm as nice looking as most people. (14) |  |  |  |  |  |
| 15. I'm pretty happy about the way I look. (15) |  |  |  |  |  |
| 16. I feel I weigh the right amount for my height. (16) |  |  |  |  |  |
| 17. I feel ashamed of how I look. (17) |  |  |  |  |  |
| 18. Weighing myself depresses me. (18) |  |  |  |  |  |
| 19. My weight makes me unhappy. (19) |  |  |  |  |  |
| 20. My looks help me to get dates. (20) |  |  |  |  |  |
| 21. I worry about the way I look. (21) |  |  |  |  |  |
| 22. I think I have a good body. (22) |  |  |  |  |  |
| 23. I'm looking as nice as I'd like to. (23) |  |  |  |  |  |

End of Block: BES

Start of Block: BSS

Q18
**The Brief Sensation Scale.**
**Instructions:**
 Please rate the extent to which you agree with the following statements.

|  | Strongly disagree (1) | Disagree (2) | Neither agree nor disagree (3) | Agree (4) | Strongly agree (5) |
| --- | --- | --- | --- | --- | --- |
| 1. I would like to explore strange places. (1) |  |  |  |  |  |
| 2. I get restless when I spend too much time at home. (2) |  |  |  |  |  |
| 3. I like to do frightening things. (3) |  |  |  |  |  |
| 4. I like wild parties. (4) |  |  |  |  |  |
| 5. I would like to take off on a trip with no pre-planned routes or timetables. (5) |  |  |  |  |  |
| 6. I prefer friends who are exctingly unpredictable. (6) |  |  |  |  |  |
| 7. I would like to try bungee jumping. (7) |  |  |  |  |  |
| 8. I would love to have new and exciting experiences even if they are illegal. (8) |  |  |  |  |  |

End of Block: BSS

Start of Block: Debrief

Q19
***Drunkorexia: Determining whether disordered eating and alcohol use is “just a university lifestyle choice.”***
 Thank you for taking part in our research study, to investigate the prevalence of compensatory behaviours in response to alcohol, in both students and non-students.
 The data collected will provide a deeper insight into an area that hasn’t really been researched outside of the student population. All answers provided from the survey will be completely anonymised and stored within the university under a password protected K Drive until the dissertation has been handed in and graded. From this, the data will be destroyed using the appropriate measures.

 We understand that reflecting on alcohol use and eating behaviours can be a sensitive topic and distressing for some people. Due to this, we have compiled a list of relevant resources and encourage you to use them if you deem it necessary:
 **Beat, UK Eating Disorder Charity** - <https://www.beateatingdisorders.org.uk/>
 **Drinkaware, Alcohol Advice** - <https://www.drinkaware.co.uk/>
 **Samaritans** - <https://www.samaritans.org/>
  
If you have any questions or concerns, please contact *Bethany Griffin at* [u1755073@unimail.hud.ac.uk](mailto:u1755073@unimail.hud.ac.uk) or their supervisor *Katharina Vogt at K.Vogt@hud.ac.uk.*

End of Block: Debrief
